# Additive Manufacturing of Poly(3-hydroxybutyrate-co-3-hydroxyhexanoate)/poly(ε-caprolactone) Blend Scaffolds for Tissue Engineering

**DOI:** 10.3390/bioengineering4020049

**Published:** 2017-05-24

**Authors:** Dario Puppi, Andrea Morelli, Federica Chiellini

**Affiliations:** BIOLab Research Group, Department of Chemistry and Industrial Chemistry, University of Pisa, UdR INSTM Pisa, via Moruzzi 13, 56124 Pisa, Italy; d.puppi@dcci.unipi.it (D.P.); a.morelli@dcci.unipi.it (A.M.)

**Keywords:** polyhydroxyalkanoates, poly(3-hydroxybutyrate-co-3-hydroxyhexanoate), poly(ε-caprolactone), polymers blend, tissue engineering, scaffolds, additive manufacturing, computer-aided wet-spinning

## Abstract

Additive manufacturing of scaffolds made of a polyhydroxyalkanoate blended with another biocompatible polymer represents a cost-effective strategy for combining the advantages of the two blend components in order to develop tailored tissue engineering approaches. The aim of this study was the development of novel poly(3-hydroxybutyrate-*co*-3-hydroxyhexanoate)/ poly(ε-caprolactone) (PHBHHx/PCL) blend scaffolds for tissue engineering by means of computer-aided wet-spinning, a hybrid additive manufacturing technique suitable for processing polyhydroxyalkanoates dissolved in organic solvents. The experimental conditions for processing tetrahydrofuran solutions containing the two polymers at different concentrations (PHBHHx/PCL weight ratio of 3:1, 2:1 or 1:1) were optimized in order to manufacture scaffolds with predefined geometry and internal porous architecture. PHBHHx/PCL scaffolds with a 3D interconnected network of macropores and a local microporosity of the polymeric matrix, as a consequence of the phase inversion process governing material solidification, were successfully fabricated. As shown by scanning electron microscopy, thermogravimetric, differential scanning calorimetric and uniaxial compressive analyses, blend composition significantly influenced the scaffold morphological, thermal and mechanical properties. In vitro biological characterization showed that the developed scaffolds were able to sustain the adhesion and proliferation of MC3T3-E1 murine preosteoblast cells. The additive manufacturing approach developed in this study, based on a polymeric solution processing method avoiding possible material degradation related to thermal treatments, could represent a powerful tool for the development of customized PHBHHx-based blend scaffolds for tissue engineering.

## 1. Introduction

Tissue engineering is a growing research area, with a few successful clinical results, aimed at developing reliable alternatives to conventional surgical strategies (e.g., auto- and allogenic tissue transplantation or artificial prosthesis implantation) for the treatment of human tissue and organ failure caused by defects, injuries or other types of damage [1]. Tissue engineering relies on the combination of cells, biomaterials and bioactive molecules to generate replacement biological tissues and organs for a wide range of medical conditions. The most common approach involves the employment of a highly porous biodegradable support, commonly referred to as the scaffold, which acts as a temporary template providing a cell adhesion substrate and mechanical support, and guiding the regeneration processes [2]. In the last two decades, a great variety of biodegradable materials and processing techniques have been investigated for the development of scaffolds with proper physico-chemical properties as well as macro-, micro- and nano-architecture features suitable for tissue growth in three dimensions [3].

Polyhydroxyalkanoates (PHAs) are microbial aliphatic polyesters widely investigated for biomedical applications due to their biodegradability and biocompatibility, as well as the wide range of mechanical and processing properties of the numerous homopolymers and copolymers belonging to this class of renewable polymers [4]. Different articles have reported on poly[(R)-3-hydroybutyrate] (PHB) and poly[(R)-3-hydroxybutyrate-*co*-(R)-3-hydroxyvalerate] (PHBV) investigations, both in vitro and in vivo, for bone tissue regeneration approaches [5,6,7]. Due to the relatively long alkyl side chain, poly[(R)-3-hydroxybutyrate-*co*-(R)-3-hydroxyhexanoate) (PHBHHx) exhibits lower crystallinity, a broader processing window and higher elasticity compared with PHB and PHBV [8]. Among the different investigated biomedical applications, PHBHHx has been proposed as scaffolding material for bone regeneration thanks to its piezoelectric behavior and cytocompatibility when cultured with osteoblasts and bone marrow cells [9,10,11,12,13,14]. In addition, recent articles showed that PHBHHx in the form of microgrooved membrane [15], aligned nanofibers [16] or carbon nanotubes-loaded composite materials [17] well supports the osteogenesis of human mesenchymal stem cells.

As defined by the American Society for Testing and Materials (ASTM), Additive Manufacturing (AM) refers to the process of joining materials to make objects from three-dimensional (3D) model data, usually layer upon layer, as opposed to subtractive manufacturing methodologies [18]. The introduction of a number of AM techniques, such as stereolithography and fused deposition modeling, into the tissue engineering field has allowed the enhancement of control over scaffold structure at different size scales (from macro- to micrometric scale) in terms of external shape and porous structure [19]. They involve a computer-controlled layered manufacturing process based on a sequential delivery of energy and/or materials starting from a 3D digital model to build up 3D polymeric scaffolds with a predefined geometry and internal porosity. Advanced computer-aided design and manufacturing approaches enable a high degree of automation, good accuracy and reproducibility for the fabrication of clinically-sized, anatomically-shaped scaffolds with a tailored porous structure characterized by a fully interconnected network of pores with customized size and shape. However, despite the promising results and widespread research on PHAs for tissue engineering applications, their narrow melt processing temperature window [20,21] has hindered the application of AM techniques for their processing into 3D porous scaffolds. Computer-aided wet-spinning (CAWS), a hybrid AM technique based on the computer-controlled deposition of a solidifying polymeric fiber extruded directly into a coagulation bath, was recently applied to process PHBHHx into 3D scaffolds with tailored geometry and networks of macropores as well as a homogenous microporous matrix [22,23].

The aim of this study was to investigate the suitability of the CAWS technique for the fabrication of scaffolds made of PHBHHx blended with poly(ε-caprolactone) (PCL). PCL is an aliphatic polyester that has been widely investigated for biomedical applications receiving FDA approval and CE Mark registration for a number of drug delivery and medical device applications [24]. Thanks to its good processing properties, tunable mechanical properties and slow biodegradation, PCL is seen as one of the most versatile scaffolding materials for the development of long-term biodegradable bone implants. Recent studies have investigated the blending of PHAs with PCL and other synthetic polyesters as a cost-effective strategy for combining the advantages of the two polymers and achieving additional desirable properties [21,25,26]. As an example, a research activity on PCL/PHBHHx blend membranes by solvent processing showed that by optimizing the weight ratio between the two components it was possible to enhance the resulting mechanical properties in comparison with PCL and PHBHHx alone [27]. Although the great versatility of the CAWS technique in customizing PHBHHx scaffold’s shape and internal architecture, in the case of inter-fiber deposition distances larger than 200 μm (i.e., 500 and 1000 μm), a well-defined porosity along the Z axis was not achieved due to the slow solidification of the coagulating fiber. On the other hand, the optimization of PCL processing by CAWS has enabled the employment of large inter-fiber deposition distances (i.e., 500 and 1000 μm) for the fabrication of 3D scaffolds with a homogeneous porosity in the cross-section characterized by a Z axis pore size in the range of hundreds of micrometers [28,29,30]. Since a pore size larger than 100 μm is recommended to achieve enhanced bone tissue regeneration and vascularization [31], blending PHBHHx with a polymer showing better processing properties was investigated during the present study as an effective strategy to develop scaffolds meeting the aforementioned structural parameters requirement. For this purpose, the CAWS conditions for the fabrication of PHBHHx/PCL scaffolds with different ratios between the two blend components were investigated. Optimized PHBHHx/PCL scaffold prototypes were characterized in comparison with PHBHHx scaffolds for their morphology by means of scanning electron microscopy (SEM) under backscattered electron imaging, thermal properties by means of thermogravimetric analysis (TGA) and differential scanning calorimetry (DSC), and mechanical properties under compression using a uniaxial testing machine. The scaffold’s biocompatibility was evaluated in vitro by employing the MC3T3-E1 murine preosteoblast cell line. Cell response, in terms of viability, proliferation and morphology was investigated by tetrazolium salts (WST-1) and confocal laser scanning microscopy (CLSM).

## 2. Materials and Methods

### 2.1. Materials

Poly(ε-caprolactone) (PCL, CAPA 6800, Mw = 80,000 g·mol^−1^) was supplied by Perstorp UK Ltd (Warrington, Cheshire, UK) and used as received. Poly(3-hydroxybutyrate-*co*-3-hydroxyhexanoate) (PHBHHx, 12% mol HHx, Mw = 300,000 g·mol^−1^) was kindly supplied by Tsinghua University (Beijing, China). PHBHHx was purified before use according to the following procedure: (i) the polymer was dissolved in 1,4 dioxane (5% *w*/*v*) under stirring at room temperature for 1 h; (ii) the solution was filtered under vacuum using filter paper; (iii) the filtrate was slowly dropped into 10-fold volume water to precipitate PHBHHx; (iv) after precipitation the polymer was collected by filtering; (v) the polymer was washed with distilled water and then ethanol, and vacuum dried and stored in a desiccator. All the solvents and chemical reagents were purchased from Sigma-Aldrich (Italy) and used as received without further purification.

### 2.2. Scaffolds Fabrication

PHBHHx solutions were prepared by dissolving the polymer in tetrahydrofuran (THF) at 32 °C under stirring for 2 h at a concentration of 25% *w*/*v*. For the preparation of PHBHHx/PCL solutions, PCL was dissolved in THF at 32 °C under stirring for 2 h and then the desired amount of PHBHHx was added to the polymer solution. The mixture was left under stirring for 2 h at 32 °C until a homogenous solution was obtained. Solutions with different PHBHHx/PCL weight ratios (3:1, 2:1 and 1:1) and a total concentration of the polymeric phase of 12% *w*/*v* were prepared.

**Figure 1:**
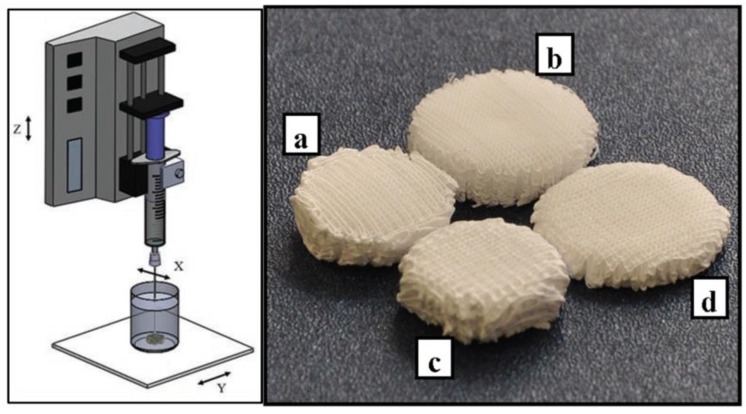
Schematics of the computer-aided wet-spinning (CAWS) process (left); representative image of the developed scaffolds (right): (**a**) PHBHHx; (**b**) PHBHHx/PCL 3:1; (**c**) PHBHHx/PCL 2:1; (**d**) PHBHHx/PCL 1:1.

Scaffolds were fabricated by means of a subtractive rapid prototyping system (MDX 40A, Roland MID EUROPE, Acquaviva Picena, Italy) modified in-house by replacing the milling head unit with a programmable syringe pump system (NE-1000; New Era Pump Systems Inc., Wantagh, NY, USA) to enable the deposition of polymeric solutions with a controlled 3D pattern (Figure 1) [29]. The 3D geometrical scaffold parameters were designed using an algorithm developed in Matlab software (The Mathworks, Inc., Natick, MA, USA). The desired polymeric solution was placed into a glass syringe fitted with a metallic needle (Gauge 23) and injected at a controlled feeding rate directly into an ethanol coagulation bath by using the syringe pump. Scaffold fabrication was carried out by employing a deposition trajectory aimed at the production of scaffolds with a 0–90° lay-down pattern, distance between fiber axis of 500 μm and layer thickness of 100 μm. The optimized initial distance between the tip of the needle and the bottom of the beaker (Z_0_) was 1.5 mm. The effect of different processing parameters, such as the deposition velocity (V_dep_) and the solution feed rate (F), on fiber collection and morphology was evaluated to produce blend scaffolds with a different PHBHHx/PCL ratio (Table 1). By employing the optimized fabrication parameters, cylindrical samples with a designed diameter of 15 mm and height of 5 mm were fabricated. The samples were removed from the coagulation bath, left under a fume hood for 24 h, placed in a vacuum chamber at about 0.5 mbar for 48 h and then stored in a desiccator for at least 72 h before characterization.

### 2.3. Morphological Characterization

The top-view and cross section (obtained by fracture in liquid nitrogen) of the scaffolds were analyzed by means of scanning electron microscopy (SEM, JEOL JSM 300, Tokyo, Japan) under backscattered electron imaging. The average fiber diameter and pore size, defined as inter-fiber distance, were measured by means of ImageJ 1.43u software on top-view micrographs with a 50X magnification. Data were calculated over 20 measurements per scaffold. 

### 2.4. Thermal Analysis

Thermal properties of the scaffolds were evaluated by means of thermogravimetric analysis (TGA) and differential scanning calorimetry (DSC). TGA was performed using TGA Q500 instruments (TA Instruments, Milano, Italy) in the temperature range 30–600 °C, at a heating rate of 10 °C/min and under a nitrogen flow of 60 mL·min^−1^. The scaffold’s thermal decomposition were evaluated by analyzing weight and derivative weight profiles as functions of temperature. DSC analysis was performed using a Mettler DSC-822 instrument (Mettler Toledo, Novate Milanese (MI), Italy) in the range −100–200 °C, at a heating rate of 10 °C/min and a cooling rate of −20 °C/min, and under a nitrogen flow of 80 mL·min^−1^. By considering the first and second heating cycle in the thermograms, glass transition temperature (T_g_) was evaluated by analyzing the inflection point, while melting temperature (T_m_) and enthalpy (∆H) was evaluated by analyzing the endothermic peaks.

### 2.5. Mechanical Testing

The scaffold’s mechanical properties were analyzed under compression using an Instron 5564 uniaxial testing machine (Instron Corporation, Norwood, MA, USA) equipped with a 2 kN load cell. After the treatment to remove residual solvents, as previously described, the samples were preconditioned at 25 °C and 50% of humidity for 48 h and then characterized at room temperature. The test was carried out on cylindrical samples with actual diameters of around 15 mm and actual heights of around 4 mm (50 layers). Six samples of each kind of scaffold were tested at a constant crosshead displacement of 0.4 mm·min^−1^ between two parallel steel plates up to 85% strain [32]. The stress was defined as the measured force divided by the total area of the apparent cross section of the scaffold, whilst the strain was evaluated as the ratio between the height variation and the initial height. Stress-strain curves were obtained from the software recording the data (Merlin, Series IX, Instron Corporation, Norwood, MA, USA). The compressive modulus was calculated as the slope of the initial linear region in the stress-strain curve, avoiding the toe region. Compressive yield strength and strain were considered at the yield point, and compressive strength was considered as the stress corresponding to 85% strain.

### 2.6. In Vitro Biological Evaluation

#### 2.6.1. Cell Culture

Mouse calvaria-derived pre-osteoblast cell line MC3T3-E1 subclone 4 was obtained from the American Type Culture Collection (ATCC CRL-2593, Manassas, VA, USA) and cultured in Alpha Minimum Essential Medium (α—MEM, Sigma, Milan, Italy) supplemented with 2 mM·L-glutamine, 10% fetal bovine serum, 100 U/mL:100 μg/mL penicillin:streptomycin solution (GIBCO, Invitrogen Corporation, Milan, Italy) and antimycotic. Before experiments, cells were trypsinized with 0.25% trypsin-EDTA (GIBCO, Gaithersburg, MD, USA) solution and resuspended in complete α-MEM at a concentration of 3 × 10^4^/mL. Scaffolds were seeded with 100 μL of cell suspension and the final volume was adjusted to 1 mL with complete medium. The specimens were then placed in an incubator with humidified atmosphere at 37 °C in 5% CO_2_. Osteogenic differentiation was induced 24 h after seeding by culturing cells in osteogenic medium prepared with α–MEM supplemented with ascorbic acid (0.3 mM) and β—glycerolphosphate (10 mM). The culture medium was replaced every 48 h and biological characterizations were carried out weekly at days 7, 14, 21 and 28. Cells grown onto tissue culture polystyrene plates were used as control.

#### 2.6.2. Cell Viability and Proliferation

Cell viability and proliferation were measured by using the (4-[3-(4-iodophenyl)-2-(4 nitrophenyl)-2H-5-tetrazolium]-1,3-benzene disulfonate) (WST-1) assay (Roche Molecular Biochemicals, Monza, Italy), which is based on the mitochondrial conversion of the tetrazolium salt WST-1 into soluble formazan in viable cells. WST-1 reagent diluted 1:10 was added to the culture and incubated for 4 h at 37 °C. Measurements of formazan dye absorbance were carried out with a Biorad microplate reader at 450 nm, with the reference wavelength at 655 nm. The in vitro biological test was performed on triplicate samples for each material.

#### 2.6.3. Morphologic Characterizations by Confocal Laser Scanning Microscopy (CLSM)

The morphology of the cells grown on the prepared meshes was investigated by means of CLSM. Cells were fixed with 3.8% paraformaldehyde in PBS 0.01 M pH 7.4 (PBS 1X), permeabilized with a PBS 1X/Triton X-100 solution (0.2%) for 15 min and incubated with a solution of 4′-6-diamidino-2-phenylindole (DAPI; Invitrogen) and phalloidin-AlexaFluor488 (Invitrogen) in PBS 1X for 60 min at room temperature in the dark. After dye incubation, samples were washed with PBS 1X before being mounted on a glass slide and sealed with resin for microscopic observation. A Nikon Eclipse TE2000 inverted microscope equipped with an EZC1 confocal laser and Differential Interference Contrast (DIC) apparatus was used to analyze the samples (Nikon, Tokyo, Japan). A 405 nm laser diode (405 nm emission) and an argon ion laser (488 nm emission) were used to excite DAPI and Alexa fluorophores, respectively. Images were captured with Nikon EZ-C1 software with identical settings for each sample. Images were further processed with GIMP (GNU Free Software Foundation) Image Manipulation Software and merged with Nikon ACT-2U software.

### 2.7. Statistical Analysis

The data are represented as mean ± standard deviation. Statistical differences were analyzed using one-way analysis of variance (ANOVA), and a Tukey test was used for post hoc analysis. A *p*-value < 0.05 was considered statistically significant.

## 3. Results and Discussion

### 3.1. Additive Manufacturing of Scaffolds

The fabrication process involved the deposition with a predefined pattern of an extruded polymeric solution into a coagulation bath to make a 3D scaffold using a layer-by-layer process. By optimizing the most influential manufacturing parameters, in terms of solution flow rate (F) and deposition velocity (V_dep_) (Table 1), 3D cylindrical scaffolds were developed by processing solutions with different PHBHHx/PCL weight ratios (3:1, 2:1 or 1:1) (Figure 1).

Wet-spinning (WS) is a non-solvent-induced phase inversion technique suitable for the industrial production of continuous polymeric fibers through an immersion-precipitation process. Briefly, a polymeric solution is injected into a coagulation bath containing a non-solvent of the polymer, and the solution filament solidifies because of polymer desolvation caused by solvent/non-solvent exchange [33]. A number of studies have shown that 3D macroporous scaffolds made of synthetic or natural polymers can be obtained through physical bonding of fibers prefabricated by means of WS, using a glue and/or a thermomechanical treatment, or in a single-step fabrication process involving the continuous, randomly-oriented deposition of the solidifying fiber by means of a manually controlled motion of the coagulation bath [33,34,35,36,37,38]. Although 3D structures with high and interconnected porosity suitable for tissue regeneration processes have been developed, an accurate control over the scaffold macro- and microstructure has not been achieved by employing these methods. The CAWS technique has been proposed as a suitable AM approach to upgrade the fabrication process in terms of reproducibility, resolution, design freedom and automation degree [39]. Advanced scaffold structural features at different scale levels, in terms of external shape and internal porosity, have been developed by applying the CAWS technique to different biocompatible polymers, including PCL, three-arm star PCL, PHBHHx, chitosan/poly(γ-glutamate) polyelectrolyte complexes and a poly(ethylene oxide terephthalate)/poly(butylene terephthalate) block copolymer [22,23,28,29,40,41,42,43,44]. As previously discussed, the application of AM to PHAs is very limited due to the narrow thermal processing window of this class of polymers. The few exceptions of PHAs scaffolds manufactured by AM are represented by a set of nanocomposite PHA/tricalcium phosphate composite scaffolds fabricated by means of selective laser sintering [45,46,47] and PCL/PHBV blend scaffolds fabricated by fused deposition modeling [21]. The research activity reported in this study has led to the development of a novel AM process for the fabrication of PHA-based blend scaffolds by processing solutions containing PHBHHx and PCL in different ratios. This approach does not require thermal treatments that could cause polymer degradation as well as denaturation of bioactive agents possibly loaded into the scaffold [48]. The solvents employed are allowed by the European Medicine Agency as residues in medical products below recommended safety levels, and classified as solvents with low toxicity (i.e., ethanol) or to be limited (i.e., THF). Considering the small volume of THF and the high ethanol/THF volume ratio involved (>20), the high volatility of the two solvents, as well as the absence of thermal events in TGA and DSC curves that could be related to the evaporation of residual solvents in scaffolds, as discussed in one of the following sections, the process can be considered as meeting the basic requirements of good manufacturing practices.

### 3.2. Morphological Characterization

**Figure 2:**
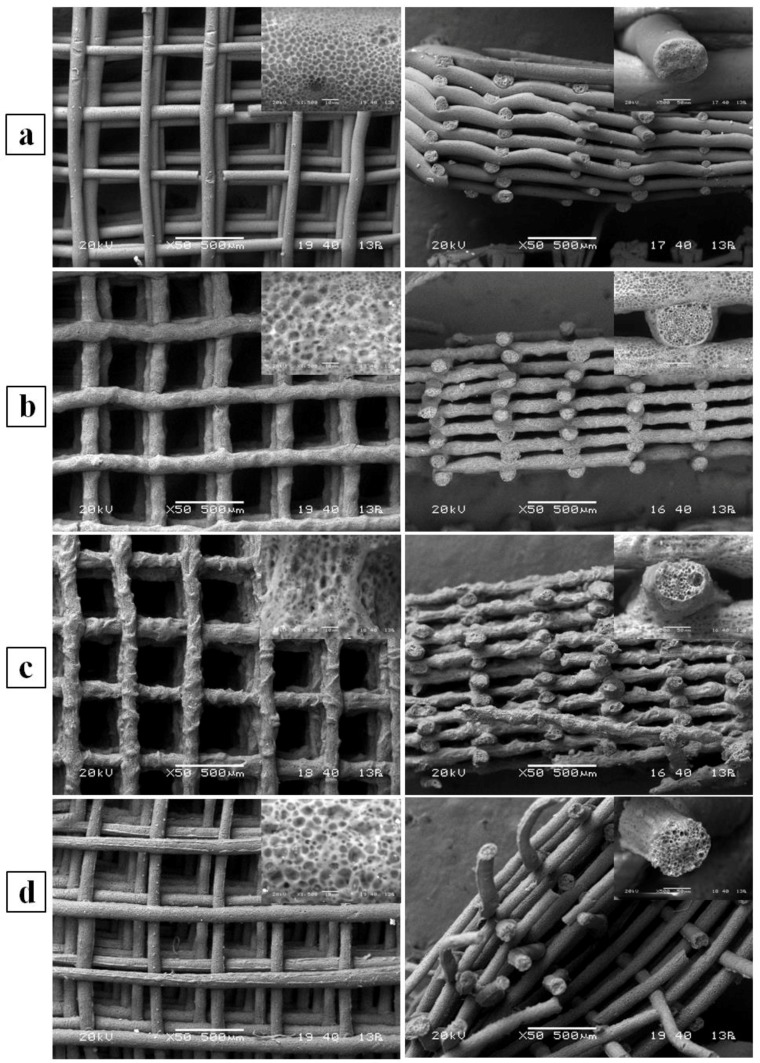
Representative top view (left) and cross-section (right) SEM micrographs of (**a**) PHBHHx; (**b**) PHBHHx/PCL 3:1, (**c**) PHBHHx/PCL 2:1, (**d**) PHBHHx/PCL 1:1. Inset high magnification micrographs show porosity of outer surface (left) and cross section (right) of single fibers.

The morphology of the developed scaffolds was investigated by means of SEM analysis using backscattering electron imaging. Analysis of samples both in top view and cross section highlighted that the fabricated scaffolds were composed by a 3D layered structure of aligned fibers forming a fully interconnected network of macropores (Figure 2). Comparative analysis of SEM micrographs showed that the scaffold’s fiber morphology and alignment were influenced by the composition of the wet-spinning solution. In addition, as observed in high magnification micrographs, the fibers had a microporous morphology both in the outer surface and in the cross-section due to the phase inversion process governing polymer solidification, as discussed elsewhere [28].

PHBHHx/PCL blend scaffolds showed significantly larger fiber diameters (average value in the range of 100 to 135 μm) in comparison with plain PHBHHx scaffolds (average value of 88 μm) (Table 1). Differences in fiber diameter among the different blend scaffolds were not statistically significant. The pore size was in the range 400–500 μm, with no statistically significant differences among the different scaffolds.

Together with the possibility of easily loading a scaffold with a drug by simply adding it to the polymeric solution before processing [41], the main advantage of CAWS is represented by the obtainment of multi-scale scaffold porosities. In fact, porous structures fabricated by means of this technique are generally characterized by a fully interconnected network of macropores, with a size that can be tuned in the range of tens to hundreds of micrometers by varying the fiber lay-down pattern, and a local micro/nanosized porous morphology of the polymeric matrix that can be tailored by acting on different parameters of the phase separation process determining polymer solidification [39]. This multi-scale morphology control represents a powerful tool to tune key scaffold properties strictly related to porosity and surface roughness, such as biodegradation rate, mechanical behavior, and cell interactions.

### 3.3. Thermal Characterization

**Figure 3:**
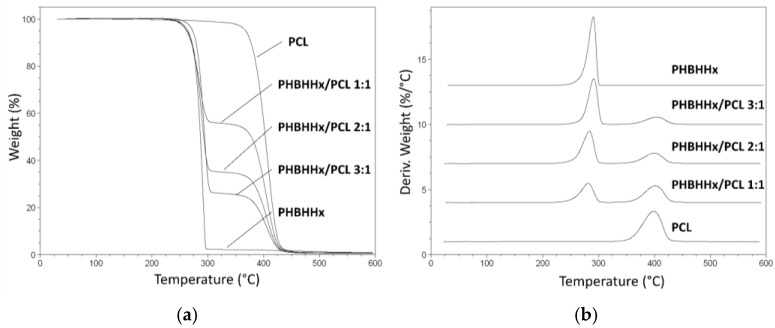
Thermogravimetric analysis (TGA) characterization: weight (**a**) and derivative weight (**b**) profiles vs temperature of the developed scaffolds.

The thermal properties of the developed PHBHHx/PCL blend scaffolds were investigated by TGA and DSC in comparison with PHBHHx scaffolds and PCL raw polymer. TGA evaluation showed that the weight and derivative weight curves of the blend scaffolds were characterized by two main thermal decomposition events: the first one centered at around 290 °C ascribable to PHBHHx decomposition, and the other one centered at around 405 °C related to PCL decomposition (Figure 3). Thermal events relevant to evaporation of residual THF (boiling point of 66 °C) or ethanol (boiling point of 78 °C) were not detected. The thermograms of the PHBHHx scaffolds are characterized by a relatively high residue at 600 °C, that did not compromise scaffold’s cytocompatibility as shown also by a previous study [22]. Nevertheless, future studies should investigate the reason and composition of this ash content and whether it may have an impact on use, particularly in extended biological testing.

The area under the first peak in derivative weight curves decreased on increasing PCL percentage, while that under the second peak increased by increasing PCL content. The resulting percentage weight losses during the first and second thermal events were close to the percentage weight in the starting polymeric solution of PHBHHx and PCL, respectively (Table 2).

**Figure 4:**
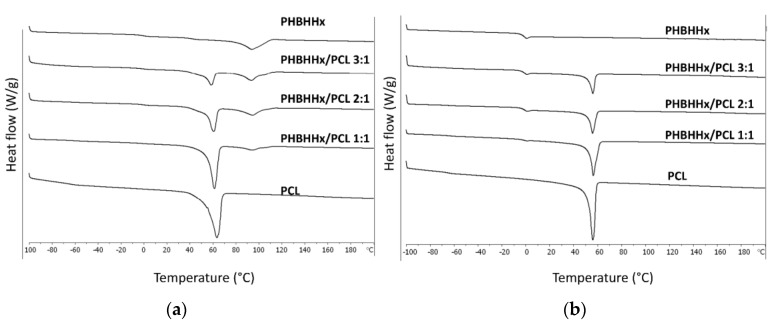
Representative differential scanning calorimetry (DSC) thermograms of the analyzed samples relevant to the first heating (**a**) and second heating (**b**) cycles.

Representative DSC thermograms of the characterized samples are reported in Figure 4. The first heating cycle analysis was carried out to assess the thermal properties of the scaffolds in comparison to what was observed in the second heating cycle after blend melting and solidification to erase the prior thermal history. The glass transition and melting temperature of PCL (Tg_1_ and Tm_1_) and PHBHHx (Tg_2_ and Tm_2_), as well as their respective melting enthalpies (ΔH_1_ and ΔH_2_), were analyzed. An endothermic peak centered at around 60 °C ascribable to the melting of PCL crystalline domains is evident in both the first and second heating cycle thermograms of blend scaffolds. The endothermic peak related to the melting of PHBHHx crystalline domains is evident only in the first heating cycle thermograms, while in the second heating cycle thermograms, a pronounced glass transition of PHBHHx only is detectable. These results corroborate what was reported in previous articles about the appreciable crystalline degree of PHBHHx scaffolds by CAWS due to the relatively slow crystallization mechanism [22,23]. Polymer crystallinity influences different properties of scaffolds made of aliphatic polyesters, such as their mechanical behavior and biodegradation rate. Indeed, as widely reported in literature [3,49], crystalline and amorphous domains show different water diffusivity as well as different macromolecular deformation and rearrangement when subjected to mechanical solicitations. The quite broad endothermic peak in the first heating scan of PHBHHx scaffolds curve can be explained with the melting of different lamellar crystalline domains formed during polymer solidification, as suggested by previous articles on thermal characterization of PHBHHx films [50,51]. Endothermic peaks related to evaporation of residual solvents were not detectable in the first nor in the second heating scan.

By comparing data from either the first or the second DSC scan (Table 3), the effect of blend composition on endothermic peaks area was quantitatively confirmed through analysis of differences in enthalpies ΔH_1_ and ΔH_2_. In addition, Tg_1_ and Tm_1_ in the first heating cycle were significantly affected by PHBHHx/PCL ratio, in agreement with a previous article showing that by increasing PHBHHx content in the blend, the melting of the PCL component was shifted to lower temperatures [27], possibly due to a slight plasticization effect of PHBHHx on the PCL phase. Besides the previously mentioned effect on ΔH_1_, differences in data from the second scan were not statistically significant [26].

### 3.4. Mechanical Characterization

**Figure 5:**
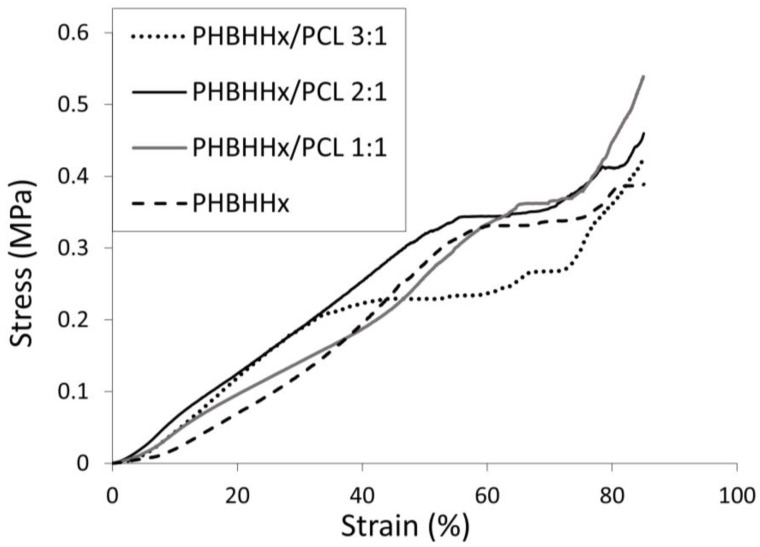
Representative stress-strain curve under compression (0.4 mm/min) of PHBHHx-based scaffolds.

The scaffold’s mechanical properties were evaluated under compression using a uniaxial testing machine at a constant strain rate. Representative stress-strain compressive curves of PHBHHx and PHBHHx/PCL blend scaffolds are reported in Figure 5. They are characterized by three distinct regions: a roughly linear region, followed by a small plateau at fairly constant stress, and a final region of steeply rising stress. As suggested by previous papers reporting on mechanical characterization of polymeric scaffolds manufactured by CAWS [22,29,41], this three-region behavior can be explained with the sample response to the applied deformation at different structural scale levels. Indeed, the linear region is likely due to the initial response of the fiber–fiber contact points, the subsequent plateau region to the collapse of the pores network, and the final stress increase region to a further densification of the scaffold structure that behaves like a dense matrix.

The characterized samples showed compressive mechanical parameters (Table 4) of the same order of magnitude of PHBHHx scaffolds previously developed by CAWS [22,23] or solvent casting combined with salt-leaching techniques [52]. PHBHHx/PCL 3:1 scaffolds showed a comparable modulus but a marked drop in yield strain, yield stress and maximum stress in comparison to PHBHHx scaffolds. However, by increasing PCL content, the compressive modulus and the other mechanical parameters increased significantly. In fact, PHBHHx/PCL 2:1 and PHBHHx/PCL 1:1 scaffolds showed compressive modulus values (0.39 ± 0.14 and 0.37 ± 0.07, respectively) between that of PHBHHx scaffolds (0.16 ± 0.12 MPa) and that of PCL scaffolds with the same designed architecture (0.60 ± 0.20 MPa) [28]. In addition, PHBHHx/PCL 2:1 scaffolds showed a yield stress (0.36 ± 0.05 MPa) comparable to that of PHBHHx scaffolds and significantly higher than the other blend scaffolds. Overall, the developed scaffolds showed lower compressive strength in comparison to PHBV/PCL blend scaffolds by fused deposition modeling [21]. This difference should be mainly related to the multi-scale porous structure of scaffolds prepared using CAWS that, although the aforementioned advantages in providing a versatile tool for tuning scaffold properties and favor cells adhesion and interactions, is characterized by a higher void volume percentage in comparison to macroporous structures with a dense polymer matrix fabricated by means of melt processing.

### 3.5. Biological Characterization

**Figure 6:**
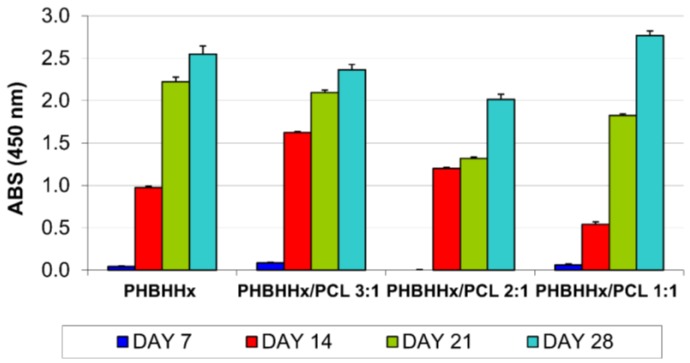
MC3T3-E1 cell proliferation on PHBHHx and PHBHHx/PCL based scaffolds.

Investigations of viability and proliferation of cells seeded onto the developed scaffolds performed using the WST-1 assay showed an increase in the number of viable cells in all the tested samples during the 28 days of culture (Figure 6).

The time-dependent changes in proliferation and differentiation can be divided into distinct stages of pre-osteoblast development [53]. The initial phase of osteoblast development is characterized by active replication of undifferentiated cells. At day 7, the observed limited cell proliferation was probably due to the large inter-fiber distance of the tested scaffolds that did not retain cells during the seeding procedure. During the second week, the cultures display a rapid increase in cell proliferation (Figure 6). The obtained data confirm a crucial role of cell–material interaction and cell density on cell adhesion, which influence cell proliferation during the initial stage of culturing [54].

**Figure 7:**
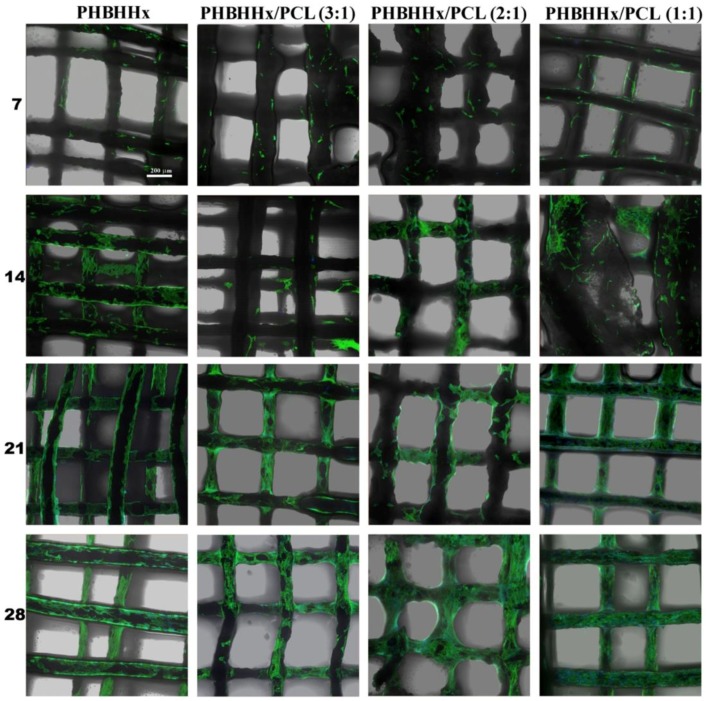
Confocal Laser Scanning Microscopy (CLSM) microphotographs showing MC3T3-E1 cell cultured on PHBHHx and PHBHHx/PCL based scaffolds, at different end-points.

Confocal Laser Scanning Microscopy (CLSM) characterization was employed to observe cell morphology on the scaffolds at different time points. Actin filament and nuclei were stained by Phalloidin-AlexaFluor488 and DAPI, and visualized as green and blue fluorescence respectively. Figure 7 shows the cell morphology and distribution of cells on the investigated samples after 7, 14, 21, and 28 days of culture. A good surface colonization of the scaffolds by MC3T3-E1 cells, with a variable shape and spreading can be observed, especially starting from the second week of culture. F-actin organization was consistent with early stages of cell adaptation to the material [55], exhibiting great stress fibers stretched along the cytoplasm, and a low cell number coherent with the quantitative proliferation data (Figure 6). Similar results for number and morphology of cells were detected by comparing different types of samples at early stages. After 14 days of culture the cell density appears to be increased, and at day 28 cells completely spread on the polymeric structure, with large cell cluster formations with inter-cellular connections (Figure 7).

These observations were in accordance with the differentiation pathway proposed for the preosteoblasts in vitro, where after an early growing latency, morpho-functional cellular aggregates are developed and single cell morphology is not distinguishable [56]. At the final phase of culturing, samples exhibited a nearly full cellular colonization of the available fiber surface by a wide continuous cell culture net.

## 5. Conclusions

The main result attained during the reported research activity is the development of an AM process based on the processing of polymeric solutions for the fabrication of PHBHHx-based blend scaffolds. This represents a novel approach to combining the advantages of PHA blending with other biocompatible polymers and the versatility of AM in supplying advanced fabrication tools for the development of scaffolds with customized macro- and microstructure. The developed manufacturing process meets both the product specification and good manufacturing practice requirements. In fact, it allows a good control of scaffold composition, external shape and internal porosity, it does not require thermal treatments that could cause material degradation, and involves the use of solvents allowed in medical device manufacturing that are completely removed from the scaffold during the fabrication and post-processing treatment. 

The characterization analyses highlighted the versatility of the developed manufacturing process by demonstrating how PHBHHx/PCL blend scaffold composition, morphological features, thermal properties and mechanical parameters could be tuned in certain ranges by varying the ratio between the two blend components in the starting solution. In addition, the results obtained from the performed preliminary biological evaluations indicated that the developed scaffolds are able to sustain a good cell adhesion and proliferation, and after 28 days of culture, scaffolds were fully colonized by MC3T3-E1 preosteoblast cells.

As shown by recent studies, the CAWS technique is well suited for the development of PHBHHx scaffolds with a complex shape resembling that of an anatomical part and a tailored porous structure with advanced architectural features at different scale levels (e.g., longitudinal macrochannel, local micro/nanoporosity) designed to enhance tissue regeneration processes [22,23]. The developed PHBHHx/PCL scaffolds can therefore represent advanced prototypes for the development of sophisticated PHAs-based blend constructs with tailored composition, anatomical shape, macroporosity and nanoporous morphology.

## Figures and Tables

**Table 1:** Optimized processing parameters, and scaffold structural parameters obtained from scanning electron microscopy (SEM) analysis.

Sample	F (mL·h^−1^)	Vdep (mm·min^−1^)	Fiber Diameter (μm)	Pore Size (μm)
PHBHHx	0.5	300	88 ± 12	485 ± 40
PHBHHx/PCL 3:1	1	560	116 ± 12	493 ± 29
PHBHHx/PCL 2:1	1	560	114 ± 15	470 ± 46
PHBHHx/PCL 1:1	1	560	120 ± 11	484 ± 18

Morphological parameters expressed as average ± standard deviation.

**Table 2:** Data relevant to thermal decomposition obtained from TGA analysis.

Sample	1st Decomposition Step		2nd Decomposition STEP	
	Peak (°C)	Weight Loss (%)	Peak (°C)	Weight Loss (%)
PHBHHx	290.7 ± 1.8	97.6 ± 0.9	-	-
PHBHHx/PCL 3:1	291.3 ± 2.2	74.6 ± 1.2	405.9 ± 0.5	25.1 ± 0.4
PHBHHx/PCL 2:1	289.2 ± 1.4	65.4 ± 1.4	405.9 ± 0.7	34.2 ± 0.5
PHBHHx/PCL 1:1	285.2 ± 1.6	44.9 ± 0.8	405.3 ± 0.8	54.6 ± 0.8
PCL raw	-	-	406.6 ± 0.4	99.1 ± 0.4

Data expressed as average ± standard deviation (*n* = 3).

**Table 3:** Data relevant to thermal characterization by DSC analysis.

Sample	1st Heating						2nd Heating			
	Tg_1_ (°C)	Tm_1_ (°C)	ΔH_1_ (J/g)	Tg_2_ (°C)	Tm_2_ (°C)	ΔH_2_ (J/g)	Tg_1_ (°C)	Tm_1_ (°C)	ΔH_1_ (J/g)	Tg_2_ (°C)
PHBHHx	---	---	---	−0.6 ± 0.2	93.7 ± 1.2	41.4 ± 1.8	---	---	---	−1.1 ± 0.6
PHBHHx/PCL 3:1	−70.7 ± 1.6	58.5 ± 0.9	18.6 ± 1.2	−0.2 ± 0.6	93.5 ± 1.6	18.4 ± 0.9	−66.2 ± 0.8	56.0 ± 1.2	15.4 ± 0.8	−1.1 ± 0.8
PHBHHx/PCL 2:1	−65.2 ± 1.4	60.7 ± 1.2	30.2 ± 1.8	−0.1 ± 0.3	94.3 ± 2.1	14.9 ± 1.1	−64.9 ± 1.3	55.8 ± 0.4	21.1 ± 1.4	−0.9 ± 0.4
PHBHHx/PCL 1:1	−60.2 ± 1.4	61.3 ± 0.8	53.4 ± 2.4	−0.6 ± 0.2	94.2 ± 1.9	7.5 ± 0.4	−63.4 ± 1.4	56.3 ± 1.6	36.0 ± 1.4	−1.4 ± 0.5
PCL raw	−61.4 ± 1.1	63.4 ± 0.5	96.5 ± 2.1	---	---	---	−64.7 ± 1.6	55.9 ± 1.5	83.0 ± 2.6	---

Data expressed as average ± standard deviation (*n* = 3).

**Table 4:** Compressive mechanical parameters of the developed scaffolds.

Scaffolds	Compressive Modulus (MPa)	Yield Strain (%)	Yield Stress (MPa)	Stress at 85% Strain (MPa)
PHBHHx	0.16 ± 0.12	56.8 ± 9.5	0.32 ± 0.02	0.47 ± 0.09
PHBHHx/PCL 3:1	0.17 ± 0.89	35.0 ± 9.4	0.18 ± 0.03	0.41 ± 0.09
PHBHHx/PCL 2:1	0.39 ± 0.14	57.9 ± 5.5	0.36 ± 0.05	0.48 ± 0.05
PHBHHx/PCL 1:1	0.37 ± 0.07	66.8 ± 5.5	0.36 ± 0.02	0.51 ± 0.07

Data expressed as average ± standard deviation (*n* = 3).
